# Fibrotic Hypersensitivity Pneumonitis: A Diagnostic Challenge Leading to Lung Transplantation

**DOI:** 10.3390/diagnostics15101267

**Published:** 2025-05-16

**Authors:** Maria-Daniela Mot, Dana Cristina Olar, Paula Alexandra Vulciu, Paula-Irina Barata, Ana-Liana Bouros-Tataru, Denis Bogdan Butari, Florin Mihai Șandor, Laura Ioana Bondar

**Affiliations:** 1 Department of General Medicine, “Vasile Goldis” Western University of Arad, Blvd. Revoluției, No. 96, 310025 Arad, Romania; mot.dana@uvvg.ro (M.-D.M.); dana.olar@student.uvvg.ro (D.C.O.); 2 Department of Biochemistry, “Vasile Goldis” Western University of Arad, Blvd. Revoluției, No. 96, 310025 Arad, Romania; vulciu.paula@uvvg.ro; 3 Department of Physiology, Faculty of Medicine, “Vasile Goldis” Western University of Arad, Blvd. Revoluției, No. 96, 310025 Arad, Romania; 4 Department of Biology and Life Sciences, Faculty of Medicine, “Vasile Goldis” Western University of Arad, Blvd. Revoluției, No. 96, 310025 Arad, Romania; tataru.liana@uvvg.ro (A.-L.B.-T.); butari.denis-bogdan@uvvg.ro (D.B.B.); sandor.florin@uvvg.ro (F.M.Ș.); bondar.lauraioana@student.uoradea.ro (L.I.B.); 5 Doctoral School of Medicine, “Vasile Goldis” Western University of Arad, Blvd. Revoluției, No. 96, 310025 Arad, Romania; 6 Doctoral School of Biomedical Sciences, University of Oradea, University Street, No. 1, 410087 Oradea, Romania

**Keywords:** antifibrotic therapy, fibrotic hypersensitivity pneumonitis, interstitial lung disease, lung fibrosis, lung transplantation

## Abstract

**Background/Objectives**: Hypersensitivity pneumonitis (HP), a subtype of interstitial lung disease (ILD), is often misdiagnosed as idiopathic pulmonary fibrosis (IPF), particularly when the causative antigen cannot be identified. Typically resulting from chronic exposure to inhaled organic particles smaller than 5 microns, HP presents a diagnostic challenge. This report outlines a case of fibrotic HP initially misclassified as asthma. No triggering antigen was identified despite extensive investigation. The disease progressed despite corticosteroid, immunosuppressive, and antifibrotic therapy, ultimately leading to an advanced fibrotic stage and requiring lung transplantation. This clinical course is rare and infrequently reported, particularly in cases requiring lung transplantation without an identifiable causative antigen. Such progression is uncommon and underreported, especially in patients initially misclassified as having asthma. **Methods**: Medical records of 24 patients diagnosed with HP were reviewed. Only one case demonstrated progression to fibrotic HP; this case was selected for detailed analysis. **Results**: Clinical and functional deterioration occurred despite standard therapy. Given the advanced stage of fibrosis and treatment resistance, lung transplantation was deemed the next appropriate therapeutic option. **Conclusions**: HP remains underdiagnosed due to difficulties in identifying the causative antigen and overlapping features with other ILDs. Early and accurate differentiation from IPF is essential, particularly in progressive fibrotic forms unresponsive to conventional therapies.

## 1. Introduction

Hypersensitivity pneumonitis (HP) is a complex and frequently underdiagnosed interstitial lung disease (ILD) resulting from an exaggerated immune response to repeated inhalation of environmental antigens [1,2]. The clinical presentation of HP is heterogeneous, encompassing acute, subacute, and chronic forms, which complicates the diagnostic process and often leads to misclassification [3,4].

Historically referred to as extrinsic allergic alveolitis, HP was first described as “farmer’s lung” in individuals exposed to moldy hay containing thermophilic actinomycetes [5,6]. Since then, a wide variety of organic and chemical antigens have been identified as potential triggers. In many cases, however, the causative agent remains elusive despite thorough investigation, contributing to diagnostic delays and progression to fibrotic disease [7,8].

**Figure 1:**
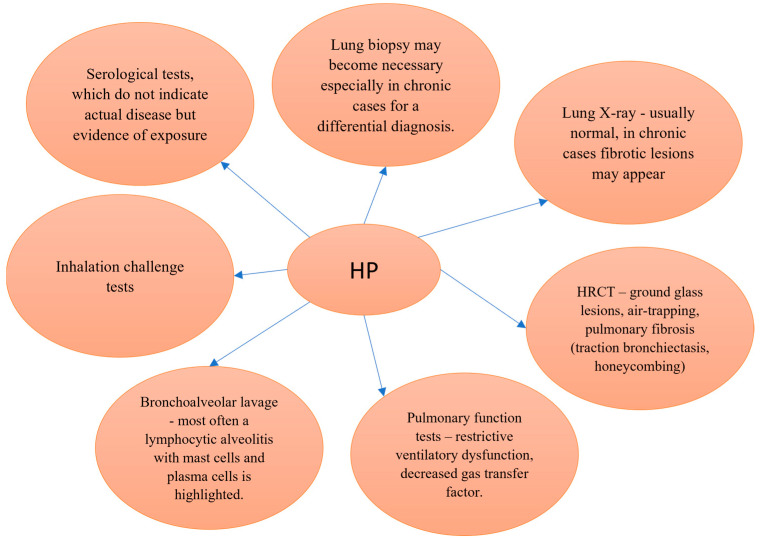
Diagnostic criteria in HP [12,13,14,15,16,17].

A major diagnostic challenge arises from the clinical, radiological, and histopathological overlap between HP and idiopathic pulmonary fibrosis (IPF) [9,10]. When the antigen cannot be identified, HP may be misdiagnosed as IPF, potentially leading to suboptimal management and worse outcomes [1,11]. Although diagnostic guidelines exist, their implementation remains variable across institutions and specialties. Figure 1 illustrates current diagnostic criteria for HP, emphasizing the importance of a multidisciplinary approach integrating clinical history, radiological features, bronchoalveolar lavage (BAL) findings, and, when necessary, histopathological confirmation [12,13,14,15,16,17].

Avoidance of antigen exposure remains the most effective strategy in managing HP. In early stages, this intervention—often coupled with corticosteroid therapy—is associated with favorable outcomes [18,19,20]. In contrast, chronic or fibrotic HP is typically less responsive to therapy and is associated with disease progression despite the use of immunosuppressive or antifibrotic agents [12,21].

Epidemiological data suggest an incidence of approximately 1 case per 100,000 individuals, though true prevalence is likely higher due to frequent misdiagnosis and underreporting [13]. Disease prognosis is highly dependent on early identification and the duration of antigen exposure. Delayed diagnosis may result in progressive lung fibrosis, reduced gas exchange capacity, and, ultimately, respiratory failure [14]. Clinical suspicion should remain high in patients with persistent cough, dyspnea, and restrictive ventilatory dysfunction, particularly when there is a potential environmental or occupational exposure history [15,16].

Recent research suggests that failure to identify a causative antigen in HP is associated with poorer prognosis and a higher likelihood of progression to fibrotic forms. Antifibrotic therapies, such as nintedanib, have shown potential in slowing disease progression in various forms of progressive fibrosing ILD, including fibrotic HP. However, therapeutic responses can be variable, and long-term outcomes remain uncertain. Furthermore, there are limited data on recurrence risk post-lung transplantation in HP, especially when the underlying trigger remains unknown [22,23,24].

Unfortunately, chronic HP tends to have a poor prognosis, often marked by progressive deterioration in general condition. Even with antifibrotic treatment and immunotherapy, the five-year survival rate appears to be around 50%, with many patients eventually requiring lung transplantation. In the case we present, a young patient showed approximately 80% lung involvement, significant impairment in functional tests, and a marked decline in quality of life. Despite extensive environmental and occupational evaluations, no antigenic trigger could be identified [25,26].

Recent studies have emphasized the challenges of differentiating fibrotic HP from IPF, especially in antigen-negative presentations [1]. Machine learning-based diagnostic tools and improved understanding of comorbid factors, such as gastroesophageal reflux disease, are reshaping diagnostic and therapeutic paradigms. However, variability in guideline implementation remains significant across institutions, contributing to underdiagnosis or misclassification. This case exemplifies these challenges and underscores the need for greater awareness of fibrotic HP evolution in atypical presentations [27,28].

Survival rates after lung transplantation for chronic HP have been reported at approximately 85% at 1 year, 74% at 3 years, and 70% at 5 years, underscoring the viability of this treatment option in advanced, therapy-resistant cases. However, an unresolved challenge remains the potential for disease recurrence in the transplanted lung, particularly when the causative antigen cannot be identified [25].

This case deserves close follow-up, as HP is included in the extended list of conditions eligible for lung transplantation, yet clinical data on such cases remain limited and sparse. No other cases of lung transplantation for this condition have been previously reported in Romania. Notably, the unknown antigen in our case raises concern about a high risk of post-transplant recurrence [25,26,29].

This case report contributes to the limited body of evidence on fibrotic HP requiring lung transplantation, particularly in cases where the inciting antigen cannot be identified. The case was selected due to its atypical progression and poor response to both immunosuppressive and antifibrotic therapies. It underscores the diagnostic uncertainties and therapeutic limitations encountered in clinical practice and highlights the need for improved recognition, earlier intervention, and more effective management strategies for fibrotic HP.

## 2. Materials and Methods

Over a seven-year period, a total of 24 patients diagnosed with HP were recorded in the clinic. Of these, 23 exhibited a favorable clinical course, either through successful elimination of the inciting antigen or corticosteroid therapy. This report focuses on the only case that progressed to a fibrotic form of HP despite medical intervention.

The patient, a male in his late 50s, initially presented with a five-month history of progressive dyspnea, cough, and fatigue. The initial clinical impression was asthma, and treatment was initiated accordingly, but no significant improvement was observed. Further investigations were conducted to explore underlying ILD and to rule out IPF.

High-resolution computed tomography (HRCT) of the chest revealed interstitial changes, including ground-glass opacities, reticulations, and features suggestive of fibrotic HP rather than IPF. Pulmonary function tests (PFTs) demonstrated a restrictive ventilatory pattern, with reduced forced vital capacity (FVC) and diffusing capacity of the lung for carbon monoxide (DLCO), findings consistent with ILD.

BAL was conducted to exclude infectious etiologies and assess inflammatory profiles. Surgical lung biopsy confirmed chronic interstitial inflammation and fibrosis, consistent with fibrotic HP, though no specific causative antigen was identified.

Initial treatment consisted of corticosteroids to reduce pulmonary inflammation, followed by immunosuppressive therapy with methotrexate. As the disease continued to progress despite therapy, antifibrotic treatment with nintedanib was initiated. However, clinical deterioration persisted, including worsening pulmonary function and increasing oxygen requirements.

Due to ongoing decline and lack of response to pharmacologic management, the patient was evaluated for lung transplantation. He met established criteria for transplant referral based on progressive, end-stage pulmonary fibrosis and respiratory failure. Due to the ongoing decline in pulmonary function and the lack of significant response to pharmacologic treatments, the patient was ultimately evaluated for lung transplantation. The decision to refer the patient for transplantation was based on several critical factors, including progressive, end-stage pulmonary fibrosis and respiratory failure, which were confirmed by clinical assessments, radiologic findings, and PFT. According to established guidelines for lung transplantation referral, the patient met the necessary criteria, including the development of irreversible functional impairment and the need for a more aggressive intervention, such as lung transplantation [30].

Informed consent was obtained from the patient for publication of this case report. All procedures and documentation adhered to institutional ethical guidelines and the Declaration of Helsinki.

## 3. Results

The case involves a male patient, currently in his late 50s, who has been followed in the clinic for over seven years. He first presented in 2017 to the emergency department of Arad Clinical Hospital with complaints of shortness of breath, cough, and fatigue, which had persisted and progressively worsened over several months. The patient reported no relevant personal or family medical history. He worked as an engineer in an environment without apparent exposure to respiratory allergens and lived in a home free of mold or animal contact. He had a 10-year history of smoking, which he discontinued at the onset of symptoms.

**Figure 2:**
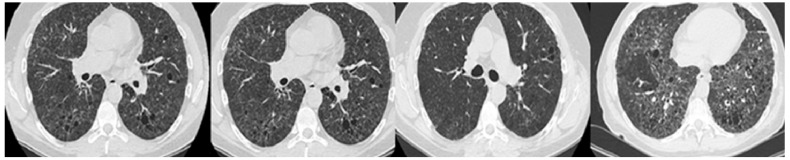
HRCT scans from 2018, 2019, 2020, and 2024, showing serial radiologic progression of chronic HP. The images demonstrate a gradual increase in mosaic attenuation, reticulation, and fibrotic changes, culminating in the development of honeycombing consistent with advanced pulmonary fibrosis.

Initial clinical examination revealed bilateral basal crepitant rales. Electrocardiogram (EKG) showed sinus rhythm and an intermediate QRS axis, with no significant abnormalities. Routine blood tests were within normal limits. Serial HRCT images obtained between 2018 and 2024 demonstrate a progressive increase in parenchymal abnormalities characteristic of chronic HP. Early scans showed diffuse centrilobular micronodules and patchy ground-glass opacities with mild mosaic attenuation. Over time, these findings evolved toward more extensive reticulation, traction bronchiectasis, and cystic changes. The most recent scan from 2024 shows clear evidence of fibrotic remodeling, with subpleural reticulation and early honeycombing, predominantly in the lower lobes—radiologic hallmarks of advanced fibrotic CHP. These changes reflect irreversible architectural distortion and suggest transition to a fibrotic phenotype with poor prognostic implications (Figure 2).

Table 1 summarizes the longitudinal progression of the patient’s pulmonary function between 2018 and 2024, with a focus on spirometric parameters and the pulmonary gas transfer factor. This comprehensive evaluation highlights the changes observed in both airflow dynamics and gas exchange efficiency over the specified period, providing valuable insights into the evolution of the patient’s pulmonary function.

To rule out autoimmune and vasculitic causes, additional serological investigations were conducted, including antinuclear antibody (ANA) testing and ImmunoCAP testing for allergies. Both tests returned negative results, as shown in Table 2 and Table 3.

Due to persistent diagnostic uncertainty, a lung biopsy was performed via left axillary mini-thoracotomy on 10 May 2019, involving atypical resections of both the left superior and inferior segments. Histological analysis revealed granulomatous pneumonitis, consistent with HP.

**Figure 3:**
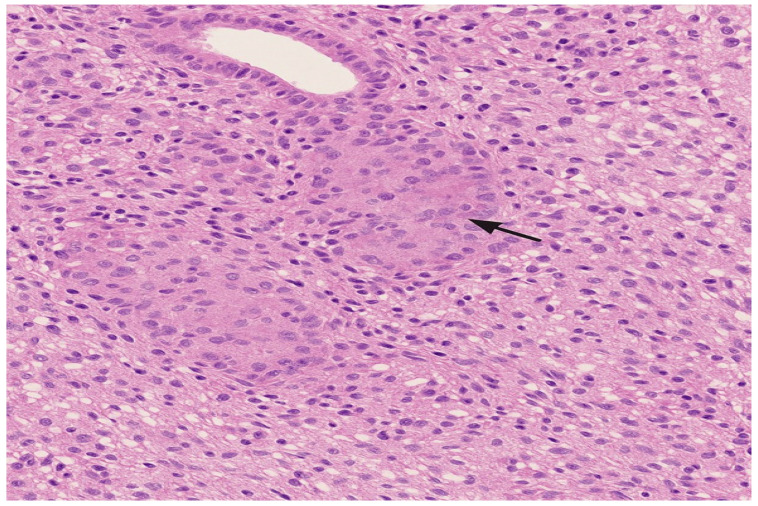
Surgical lung biopsy showing poorly formed, non-necrotizing granulomas with peribronchiolar distribution (arrow). H&E stain, 100× magnification.

Histopathological analysis revealed non-necrotizing, poorly formed granulomas with a predominantly peribronchiolar and interstitial distribution. Associated findings included chronic bronchiolocentric inflammation, alveolar septal thickening, and mild interstitial fibrosis, consistent with fibrotic HP (Figure 3). To exclude alternative diagnoses, immunohistochemical staining was performed. CD1a and Langerin were negative, ruling out Langerhans cell histiocytosis. No features of desquamative interstitial pneumonia (DIP) or respiratory bronchiolitis-associated ILD (RB-ILD) were present. These findings, integrated with radiological and clinical data, supported the diagnosis of chronic fibrotic HP.

Following the confirmed diagnosis, an extensive re-evaluation of the patient’s occupational and residential environments was undertaken, but no antigenic trigger could be identified despite changes in both work and home settings.

Immunosuppressive therapy with mycophenolate mofetil (500 mg twice daily) and corticosteroids was initiated. Despite treatment, the patient experienced progressive deterioration, including oxygen desaturation (SaO_2_ 91%), fatigue, and marked asthenia. New spirometric changes emerged by 2020, summarized in Table 4.

In 2022, due to continued clinical deterioration and progressive fibrotic changes on imaging, a diagnosis of chronic fibrotic HP was established. Antifibrotic therapy with nintedanib (150 mg twice daily) was initiated. The patient experienced mild symptomatic improvement over the first year of treatment.

By December 2024, follow-up evaluations showed further disease progression, with HRCT revealing worsening fibrosis and mixed parenchymal changes (Figure 2). Gas exchange significantly declined (DLCO 32%), and lung volume measurements indicated reduced RV (49%) and TLC (75%) (Table 1).

**Figure 4:**
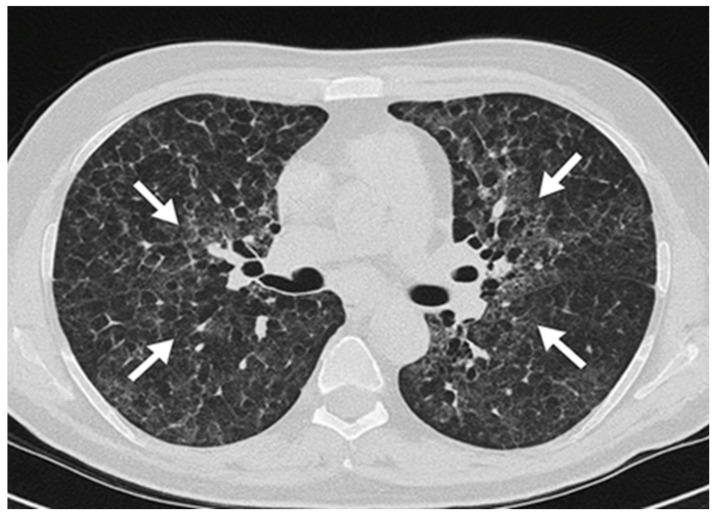
Expiratory HRCT demonstrating areas of air trapping (arrows), indicative of small airway involvement consistent with chronic HP.

As part of the radiological work-up, both inspiratory and expiratory HRCT scans were performed. Expiratory imaging revealed the presence of air trapping, which supported the diagnosis of chronic fibrotic HP (Figure 4). These findings were consistent with small airway involvement, a common feature in chronic HP.

Arterial blood gas (ABG) analysis revealed hypoxemia (PaO_2_ 61 mmHg on room air), with a pH of 7.43 and PaCO_2_ of 36 mmHg, consistent with impaired gas exchange in advanced fibrotic lung disease (Table 4).

Ongoing clinical and functional decline, despite optimized medical therapy, led to referral to a specialized lung transplantation center for advanced care evaluation.

## 4. Discussion

Over a period of seven years, 21 cases of HP were identified in the department. Most cases presented in the acute or subacute stage and responded well to antigen avoidance or corticosteroid therapy. This case was selected for detailed presentation due to its atypical course, in which no causative agent could be identified, and the disease progressed to a fibrotic form despite comprehensive evaluation and treatment.

Diagnosing HP remains a clinical challenge due to its nonspecific symptomatology and radiological overlap with other ILDs. In this case, initial imaging findings and the patient’s smoking history led to a differential diagnosis of Langerhans cell histiocytosis, illustrating how easily HP may be misclassified. Additionally, the presence of cystic lung changes in HP is associated with a worse prognosis, further complicating early identification and management [31,32,33].

Multidisciplinary discussion (MDD) was pivotal in reaching the diagnosis. MDD, involving pulmonologists, radiologists, and pathologists, is now considered the gold standard for ILD diagnosis, especially in cases with overlapping or ambiguous findings [34,35]. This collaborative approach was critical, especially in the absence of a clear environmental or occupational antigen exposure.

Cystic and emphysematous changes observed in this case are not uncommon in fibrotic HP. These radiological findings likely result from a combination of small airway obstruction, traction bronchiolectasis due to peribronchiolar fibrosis, and air trapping. Chronic antigen exposure and bronchiolar inflammation may also contribute to cyst formation. In fibrotic HP, such changes tend to have a centrilobular or peribronchiolar distribution, sometimes mimicking the appearance of honeycombing or smoking-related emphysema, even in non-smokers. Their presence can complicate the radiologic differential diagnosis, particularly in distinguishing fibrotic HP from IPF, which typically presents with subpleural honeycombing, or from Langerhans cell histiocytosis (LCH) in smokers. Moreover, extensive cystic changes may be associated with increased risk of pneumothorax and poorer outcomes and may alter the trajectory of disease by contributing to ventilatory inefficiency and gas exchange abnormalities [36].

Due to the lack of a definitive diagnosis, along with the nonspecific symptoms and history of the patient, we decided to perform specific IgE testing (ImmunoCAP) to exclude an active type I allergic reaction, which returned negative results. However, IgE testing was not useful in diagnosing HP, as this condition is mediated by a type IV immunologic mechanism involving T cell responses and IgG antibodies, rather than IgE. IgG testing for various inhaled stimuli was also performed in an attempt to identify a causative antigen, but all results were negative, with no specific agent detected as contributing to the development of HP [37].

The identification of air trapping on expiratory HRCT served as a key radiologic feature supporting the diagnosis of chronic HP. This finding reflects functional impairment of the small airways, which is frequently observed in chronic HP due to bronchiolar inflammation and fibrosis. As such, the evaluation of expiratory imaging is essential in suspected cases, as it allows for the detection of subtle small airway disease that may not be apparent on inspiratory scans alone [38].

BAL analysis was performed to evaluate the cellular inflammatory profile and rule out infection. The total cell count was within normal limits, with a differential cell count revealing approximately 10% lymphocytes, 4% neutrophils, 1% eosinophils, and 85% macrophages, indicating the absence of BAL lymphocytosis. These findings align with the advanced fibrotic stage of HP, in which lymphocyte predominance typically decreases due to ongoing fibrosis and architectural lung distortion [39,40].

Although a mildly reduced FEV1/FVC ratio and markedly decreased MEF25 (54%) may suggest small airway involvement, the lung volumes did not display features typical of obstructive physiology. TLC was reduced (75% predicted), residual volume (RV) was decreased (49% predicted), and the RV/TLC ratio was also below the threshold typically associated with air trapping (62%). The elevated ERV (177% predicted) and low DLCO (32% predicted) are consistent with fibrotic ILD. Taken together, the data support a restrictive ventilatory defect with possible early small airway involvement—likely due to bronchiolar fibrosis—rather than overt obstructive disease [41].

An apparent improvement in FVC (from 79% to 98%) was noted during follow-up, despite worsening of diffusion capacity (DLCO from 42% to 32%) and HRCT findings. This discordance may be attributed to several factors, including inter-test variability, patient effort, and a potential transient reduction in inflammation or airway resistance following immunosuppressive therapy. However, this increase in FVC does not necessarily reflect disease regression. In fibrotic HP and other ILDs, FVC can occasionally improve or stabilize even as fibrosis progresses, especially when DLCO and imaging findings show decline. This highlights the importance of a multimodal approach in disease monitoring, integrating functional, radiological, and clinical parameters [42].

This case was evaluated using the ATS/JRS/ALAT Clinical Practice Guideline for the diagnosis of HP in adults. According to this guideline, the patient met the criteria for a diagnosis of fibrotic HP with moderate diagnostic confidence [40,43].

The HRCT revealed reticulation, centrilobular nodules, and areas of air trapping on expiratory imaging, without a definitive upper lobe predominance or mosaic attenuation, findings consistent with a probable fibrotic HP pattern. Histopathological examination of the lung biopsy showed poorly formed, non-necrotizing granulomas with a peribronchiolar distribution and associated bronchiolocentric fibrosis, which aligns with the histologic pattern of probable HP [44]. Although BAL analysis did not demonstrate lymphocytosis, this absence does not exclude the diagnosis in fibrotic stages of HP, where lymphocyte predominance may diminish due to ongoing fibrosis.

The relatively low dose used in this case may have contributed to suboptimal immunosuppression and inadequate disease control, potentially influencing the progression to advanced fibrosis. This underscores the importance of early dose optimization and close monitoring of treatment response in patients with suspected fibrotic HP. The initial low dose of mycophenolate mofetil (500 mg twice daily) was selected based on a cautious approach, taking into account the patient’s general condition, risk of adverse effects, and potential gastrointestinal or hematologic intolerance. This strategy is often employed when initiating immunosuppression in patients with comorbidities or uncertain tolerance, with plans for dose escalation based on clinical and functional response. Unfortunately, in this case, disease progression outpaced the opportunity for titration to higher therapeutic levels [45].

The absence of a clearly identified antigen in this case may be explained by the “two-hit” hypothesis of HP pathogenesis. This model suggests that both a genetic predisposition and environmental exposure are required to initiate disease. Not all individuals exposed to common antigens will develop HP, highlighting the role of host susceptibility and immune response variability [3,46].

The patient was closely monitored throughout the COVID-19 pandemic. Notably, although he contracted SARS-CoV-2 in 2022, no acute exacerbation of his underlying condition occurred. This contrasts with reports indicating that COVID-19 may exacerbate pre-existing ILDs, often requiring increased corticosteroid use [47,48].

Lung transplantation remains the definitive treatment option for patients with advanced fibrotic HP and progressive respiratory failure. Although considered a last resort, transplantation offers improved quality of life and survival in appropriately selected candidates [49].

During the pandemic, the patient also participated in a pulmonary rehabilitation program targeting individuals with pulmonary fibrosis and chronic obstructive pulmonary disease (COPD). However, no measurable clinical benefit was observed in his case [50].

The use of antifibrotic therapy in HP remains an evolving area of investigation. Nintedanib, approved for use in progressive fibrosing ILDs based on the results of the INBUILD trial, has demonstrated a capacity to slow disease progression in patients with non-IPF fibrosis, including fibrotic HP [51,52]. Nonetheless, treatment response is heterogeneous, and real-world effectiveness may vary depending on the stage of disease and radiological phenotype. In this case, symptom stabilization was observed initially, followed by further decline, supporting the need for individualized treatment strategies.

Although the patient’s clinical course fulfills the criteria for progressive pulmonary fibrosis (PPF) as outlined in recent international guidelines, the progression was considered atypical due to its rapidity, severity, and resistance to both immunosuppressive and antifibrotic therapy. Notably, no triggering antigen could be identified despite thorough investigation, further complicating disease management. This unusually aggressive course highlights the heterogeneity of fibrotic HP and the need for individualized therapeutic strategies, including early consideration of lung transplantation in select cases.

It is important to acknowledge that the initial dose of mycophenolate mofetil (500 mg twice daily) administered in this case may be considered subtherapeutic based on current clinical practice guidelines and published experience in fibrotic ILD. Therapeutic doses typically range from 1500 to 3000 mg/day, with many centers initiating treatment at 1000 mg twice daily, particularly in patients with progressive fibrosing phenotypes [53,54].

This case report is limited by its single-patient design and the absence of genetic or molecular testing, which may have helped identify predispositions associated with fibrotic progression. Additionally, although a thorough environmental and occupational history was taken, the lack of an identifiable antigen remains a diagnostic challenge common to many HP cases. Future studies should explore the utility of genomic and exposomic profiling to better stratify patients with chronic HP.

From a clinical perspective, this case underscores the importance of considering fibrotic HP in the differential diagnosis of cystic lung disease, even in the absence of known exposures. It also reinforces the value of a multidisciplinary approach, timely histologic confirmation, and individualized therapy planning. Given the variable response to antifibrotic agents such as nintedanib, there remains a need for robust, HP-specific clinical trials to guide therapeutic decision-making in progressive fibrosing phenotypes.

This case presents an atypical progression of HP, where no causative antigen was identified, and the disease advanced to fibrosis despite standard treatments. Unlike most cases of HP, which respond well to antigen avoidance or corticosteroid therapy, this patient’s disease continued to progress, highlighting the potential for a more aggressive, unresponsive form of HP.

The novelty of this case lies in its rapid progression to fibrosis without a clear antigenic trigger, contrasting with the typical course of HP. While immunosuppressive therapies such as corticosteroids and mycophenolate mofetil are often effective, this patient’s lack of response suggests that alternative treatments or early lung transplantation may be necessary in cases of severe, progressive HP.

This case contributes to the literature by underscoring the variable nature of HP and the need for individualized treatment strategies, especially in patients who do not respond to conventional therapies. Further research is required to explore effective management options for fibrotic HP and to identify predictors of disease progression [55].

## 5. Conclusions

This case highlights the diagnostic and therapeutic complexities associated with fibrotic HP, particularly when no causative antigen can be identified. The nonspecific nature of clinical symptoms, overlap with other ILD, and the presence of cystic changes on imaging contributed to delayed diagnosis and initial misclassification. Despite a comprehensive evaluation, including MMD, histopathological confirmation, and BAL, the disease progressed to an advanced fibrotic stage.

Management included corticosteroid therapy, immunosuppressive agents, and antifibrotic treatment with nintedanib, yet the patient continued to deteriorate, ultimately necessitating referral for lung transplantation. This case underscores the importance of early recognition, accurate phenotypic classification (fibrotic vs. non-fibrotic), and individualized therapeutic strategies to potentially improve prognosis and delay disease progression.

The patient’s trajectory also reinforces the relevance of the “two-hit” hypothesis, which posits that genetic susceptibility and environmental exposures must coexist to initiate the disease. While this model offers a valuable framework for understanding HP pathogenesis, the inability to identify an antigen in this case raises important questions about the role of subclinical exposures, threshold effects, or unidentified genetic predispositions.

From a clinical standpoint, this case advocates for increased awareness of atypical presentations of HP and the need for prompt referral to specialized centers. Additionally, it emphasizes the critical role of multidisciplinary collaboration in guiding diagnosis and treatment. Future research should focus on identifying reliable biomarkers, refining environmental exposure assessment tools, and expanding evidence-based treatment options, particularly for patients with progressive fibrosing phenotypes who may not respond adequately to current therapies.

## Figures and Tables

**Table 1:** Chronological Evolution of Pulmonary Function Parameters.

Diffusion Capacity and Lung Volumes/Pulmonary Function Test						
Data	FVC %	DLCO mmol/(min*kPa)	TLC L	RV/TLC %	ERV %	RV %
2018	80	42	85	70	185	58
2020	78	38	75	50	179	55
2022	109	36	74	38	191	50
2024	93	32	75	62	177	49

FVC = Forced Vital Capacity; DLCO = Diffusing capacity of the lung for carbon monoxide, expressed in mmol/(min*kPa); TLC = Total Lung Capacity, expressed in liters; RV/TLC = Residual Volume to Total Lung Capacity ratio, expressed as a percentage; ERV = Expiratory Reserve Volume, expressed as a percentage; RV = Residual Volume, expressed as a percentage.

**Table 2:** ANA IgG Antibody Panel Results.

Antibody	Result	Reference Value
Anti-nRNP/Sm	Negative	(Negative)
Anti-Sm	Negative	(Negative)
Anti-SS-A (Ro)	Negative	(Negative)
Anti-Ro-52	Negative	(Negative)
Anti-SS-B (La)	Negative	(Negative)
Anti-Scl-70	Negative	(Negative)
Anti-PM-Scl 100	Negative	(Negative)
Anti-Jo-1	Negative	(Negative)
Anti-Centromere B	Negative	(Negative)
Anti-PCNA	Negative	(Negative)
Anti-dsDNA	Negative	(Negative)
Anti-Nucleosome	Negative	(Negative)
Anti-Histone	Negative	(Negative)
Anti-Ribosomal P Protein	Negative	(Negative)
Anti-AMA-M2	Negative	(Negative)
Anti-DFS70	Negative	(Negative)

**Table 3:** ImmunoCAP Allergy Test Results and CAP-Class Interpretation.

CAP-Class Reference Ranges		
CAP-Class	AU/mL Range	Interpretation
Class 0	0.10–0.35	Negative
Class 1	0.35–0.70	Threshold value
Class 2	0.70–3.50	Weakly positive
Class 3	3.50–17.5	Positive
Class 4	17.5–50.0	Highly positive
Class 5	50.0–100	Intensely positive
Class 6	>100	Exceptionally high value
**Allergen-Specific IgE Results with CAP-Class Interpretation**		
**Allergen**	**Result (AU/mL)**	**CAP-Class**
Chicken feathers	<0.10 (Negative)	Class 0
Chicken droppings	<0.10 (Negative)	Class 0

Notes: This ImmunoCAP test demonstrates high sensitivity, detecting allergen-specific IgE levels from 0.1 to 0.34 kU/L. Results below 0.35 kU/L are typically interpreted as negative. However, in rare cases, low-level background reactivity may occur without clinical relevance.

**Table 4:** ABG Analysis Indicating Gas Exchange Impairment.

Parameter	Value (On Room Air)
pH	7.43
PaO_2_	61 mmHg
PaCO_2_	36 mmHg
HCO_3_^−^	23 mEq/L
SaO_2_	90%

pH = Potential of hydrogen (measure of blood acidity/alkalinity); PaO_2_ = Partial pressure of oxygen in arterial blood; PaCO_2_ = Partial pressure of carbon dioxide in arterial blood; HCO_3_^−^ = Bicarbonate concentration in arterial blood; SaO_2_ = Arterial oxygen saturation (percentage of hemoglobin bound to oxygen).

## Data Availability

The data presented in this study are available on request from the corresponding author.

## Abbreviations

The following abbreviations are used in this manuscript:

HP	Hypersensitivity Pneumonitis
HRCT	High-Resolution Computed Tomography
ILD	Interstitial Lung Disease
IPF	Idiopathic Pulmonary Fibrosis
PFT	Pulmonary Function Test
FVC	Forced Vital Capacity
DLCO	Diffusing Capacity of the Lung for Carbon Monoxide
BAL	Bronchoalveolar Lavage
EKG	Electrocardiogram
ANA	Anti-Nuclear-Antigen antibody panel
IgG	Immunoglobulin G
FEV	Forced Expiratory Volume
TLC	Total Lung Capacity
RV	Residual Volume
IgE	Immunoglobulin E
COPD	Chronic Obstructive Pulmonary Disease
ERV	Expiratory Reserve Volume
RB-ILD	Respiratory Bronchiolitis-Associated Interstitial Lung Disease
PaO_2_	Partial Pressure of Oxygen
HE	Hematoxylin and Eosin
PaCO_2_	Partial Pressure of Carbon Dioxide
PPF	Progressive Pulmonary Fibrosis
ABG	Arterial Blood Gas
MDD	Multidisciplinary discussion
ATS	American Thoracic Society
JRS	Japanese Respiratory Society
ALAT	Latin American Thoracic Association
Anti-nRNP/Sm	Anti-nuclear Ribonucleoprotein/Smith antibodies
Anti-Sm	Anti-Smith antibodies
Anti-SS-A (Ro)	Anti-SS-A (Ro) antibodies
Anti-Ro-52	Anti-Ro-52 antibodies
Anti-SS-B (La)	Anti-SS-B (La) antibodies
Anti-Scl-70	Anti-topoisomerase I (Scl-70) antibodies
Anti-PM-Scl 100	Anti-PM-Scl 100 antibodies
Anti-Jo-1	Anti-histidyl-tRNA synthetase (Jo-1) antibodies
Anti-Centromere B	Anti-centromere B antibodies
Anti-PCNA	Anti-proliferating cell nuclear antigen (PCNA) antibodies
Anti-dsDNA	Anti-double-stranded DNA antibodies
Anti-Nucleosome	Anti-nucleosome antibodies
Anti-Histone	Anti-histone antibodies
Anti-Ribosomal P Protein	Anti-ribosomal P protein antibodies
Anti-AMA-M2	Anti-mitochondrial antibodies type M2
Anti-DFS70	Anti-dense fine speckled 70 antibodies
DIP	Desquamative Interstitial Pneumonia
SaO_2_	Arterial Oxygen Saturation
HCO_3_−	Bicarbonate
MEF25	Maximal Expiratory Flow at 25% of Forced Vital Capacity
